# Open chemistry: RESTful web APIs, JSON, NWChem and the modern web application

**DOI:** 10.1186/s13321-017-0241-z

**Published:** 2017-10-30

**Authors:** Marcus D. Hanwell, Wibe A. de Jong, Christopher J. Harris

**Affiliations:** 1 Kitware, Inc., 28 Corporate Drive, Clifton Park, NY 12065 USA; 2 LBNL, One Cyclotron Road, Berkeley, CA 94720 USA

**Keywords:** Chemistry, Web, Data, Semantic, NWChem, JSON

## Abstract

An end-to-end platform for chemical science research has been developed that integrates data from computational and experimental approaches through a modern web-based interface. The platform offers an interactive visualization and analytics environment that functions well on mobile, laptop and desktop devices. It offers pragmatic solutions to ensure that large and complex data sets are more accessible. Existing desktop applications/frameworks were extended to integrate with high-performance computing resources, and offer command-line tools to automate interaction—connecting distributed teams to this software platform on their own terms. The platform was developed openly, and all source code hosted on the GitHub platform with automated deployment possible using Ansible coupled with standard Ubuntu-based machine images deployed to cloud machines. The platform is designed to enable teams to reap the benefits of the connected web—going beyond what conventional search and analytics platforms offer in this area. It also has the goal of offering federated instances, that can be customized to the sites/research performed. Data gets stored using JSON, extending upon previous approaches using XML, building structures that support computational chemistry calculations. These structures were developed to make it easy to process data across different languages, and send data to a JavaScript-based web client.

## Background

The in-silico determination of chemical and materials properties is a vital capability that drives innovation across many market sectors. Its importance is reflected in the number of codes that can perform simulations over a broad range of levels of theory and length scales [1–5], and the enormous investments in experimental facilities that can also produce large data that is often difficult or impossible to reproduce. However, it is all too common for experimental and computational studies to take place independently, with large scale studies often involving heroic efforts developing one-off software projects dedicated to the specific resource, such as the Protein Data Bank [6] (over 100,000 experimental structures), Materials Project [7] (over 33,000 simulated materials), and the Clean Energy Project [8] (over 2.3 million calculated structures).

Driven by the U.S. Materials Genome Initiative the development of new and novel materials has become a multidisciplinary research endeavor where complex simulation and experimental data get integrated, and analytics such as machine learning techniques are utilized to aid in scientific discovery. This same multidisciplinary approach is becoming essential in chemical and biological research and development, in the design of new chemicals, biomolecules and drugs, or new energy efficient chemical production processes.

There is a strong need to develop a collaborative scientific research software platform that enables researchers to define concepts and hypotheses, add them, and analyze integrated sets of experimental and computational data to offer effective knowledge discovery more universally. This must go well beyond a web portal to create an interactive platform integrating simulation, experimental data, and analytics, while leveraging semantic web technologies to support the federated storage of data across geographically dispersed sites. The use of language independent programming interfaces that are consumed by web, desktop, and command-line clients will enable researchers to make much better use of their data. The platform described aims to provide an open source service that can satisfy this basic need, and also offer simple methods of extension to accommodate new areas of research.

The web has evolved significantly in the last two decades, and new developments should be explored to assess what can be achieved in a platform that seeks to use the latest open source tools, technologies, standards, and approaches to deliver an end-to-end platform for chemical/materials research. The basic approach employed was to develop a server component written in the Python language that exposes RESTful (representational state transfer) endpoints to interact with the data on the server. The Python code can use existing core functionality, Python modules, and wrapped libraries developed in other programming languages.

Ideally no HTML, images, etc would be generated on the server, the server acts as a data server primarily through RESTful endpoints that accept/return data, along with user authentication (required by some endpoints). It can trigger calculations, perform analyses, and batch jobs in order to make the data discoverable. The server can then be consumed by a rich HTML5 web client, more traditional desktop clients, and from command-line clients or other servers to perform automated workflows.

In this work a rich HTML5 web interface was developed that made use of the server’s API (application programming interface) briefly discussed above. It consumes data from the server, uploading/editing data, and maintaining local state in a one-page application reusing a popular open source HTML5 framework. The application integrated other open source frameworks for client side charting, and 3D rendering/visualization of molecular structure.

The web application was developed using open source tools, a number of frameworks, and was “built” as a static bundle of HTML5 web assets that are downloaded by the web client. It dynamically constructs the page in response to user interaction, server data, and other client events to provide a rich, interactive experience. This means that many interactions take place entirely on the client, requiring no interaction with or access to the server. This offers interactive data visualization and analysis, even on relatively low bandwidth links, once initial data for a molecule or calculation has been downloaded.

The field of chemical sciences needs access to open source chemical data services that make use of open data standards and formats. The development of tools with programming language agnostic interfaces available over standard web protocols is described. Standardized data representations in the database layer were employed, with translation facilities using existing open source tools to existing file formats, along with integrated visualization/analysis capabilities in the web browser.

The project can be used directly, extended for related use cases, or built upon in future work to offer a more comprehensive semantically enriched platform for chemical data. Most existing platforms use approaches that mix data with web page generation on the server, rather than embracing modern client-server approaches using the latest web standards and frameworks. The source code is often not available, and the platforms are curated centrally—such as the PDB, Materials Project and Clean Energy Project mentioned earlier.

## Methods

**Fig. 1:**
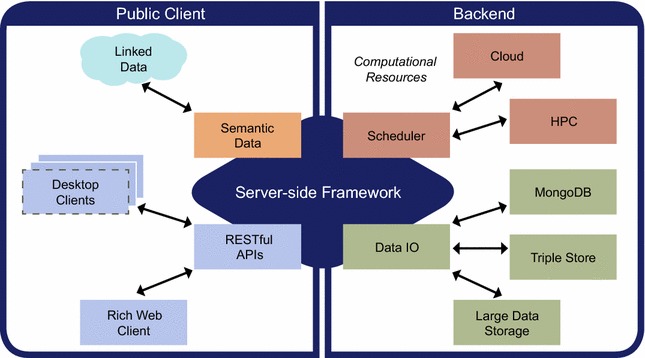
Architecture of the chemical data platform. Overview of the high-level architecture used for the chemical data platform

An open source prototype web platform was developed to demonstrate key capabilities in addressing the needs outlined in the introduction, and summarized in Fig. 1. The application has a number of components developed in several languages following modern development methodologies. It was intentionally developed using some of the latest technology innovations, which means that it requires a modern web browser that supports WebGL [9] in order to render 3D geometry, and it makes extensive use of HTML5 [10]. It is clear that not all devices/web browsers have full support for these technologies at this time, but that this support is already substantial and will grow in the coming years.

The server-side components were written in Python [11], with wrapped C++ code [12] providing access to a number of existing chemistry/materials libraries such as Avogadro [13] and Open Babel [14]. The basis for the server-side project was the Girder project [15], which reuses a number of Python modules such as CherryPy [16] to provide a minimalist Python web framework, Swagger [17] to document the programming interfaces, MongoDB [18] to store data/metadata, and Virtuoso [19] to store triples. The chemistry specific functionality was developed as additional programming interfaces using the Girder project’s plugin mechanism to add additional endpoints extending upon existing functionality.

The client-side components were developed in HTML5, using open source web frameworks/technologies such as AngularJS 1.6 [20] and Material Design [21] to provide a single page web application. 3DMol.js [22] was used to render molecular geometry in 3D, D3 [23] to render charts, and responsive design elements to accommodate devices of various sizes/aspect rations. The capabilities developed have been demonstrated on desktop browsers, mobile phones, and tablets on the major operating systems. This includes Windows, macOS, Linux, iOS, and Android operating systems using browsers including Chrome, Firefox and Safari.

The features developed for the web platform made it possible to expose functions originally developed for command-line/desktop use, this led to the reuse of the Avogadro 2 libraries [24] for the ingestion of chemical data. Extensions to the Avogadro 2 libraries, and several other components, were made to support the web platform. These have been merged into the main development branch, and were made available in the 1.90 release of the software. Capabilities, such as the visualization/animation of vibrational data, and additional file formats supporting the NWChem package were added to the library. The JSON readers/writers built upon the JsonCpp library [25], and the capabilities were exposed to the Python-based server using Boost.Python [26] wrapped calls to the C++ API, and then mapped to web endpoints in the Python code developed for the server.

The computational chemistry code used as a generator for the calculation results is the open source NWChem software suite [1]. The JSON-Fortran library [27] was integrated into the NWChem source to enable the code to write out a new JSON file in addition to the standard output or log file. APIs and interfaces between the Fortran-90 routines of the JSON-Fortran library and the Fortran-77 NWChem source were written to facilitate the transfer of the computational chemistry data into the JSON format. The full JSON enabled NWChem source code is available on Github [28]. To enable the end-user to convert existing log files to the JSON format a Python 3 library was created. The library and examples are available in a separate Github repository [29].

New JSON data structures were developed to support the end-to-end workflow from data generation, through to ingestion, analysis, and visualization. Python scripts were also developed to upload and add data files from the command-line, enabling ingestion of existing data sets, and new ones as they are generated.

## Results

The software components were developed to serve the needs of data-centric chemistry research using open source approaches that embrace the use of open APIs, open data formats, and open components. The approach made extensive use of client-side rendering/interaction wherever practical, and focused on a server-side component that served data from web endpoints using the web-native JSON format where possible. The development spanned a number of programming languages (Fortran, C++, Python, and JavaScript) in order to offer structured data that can be stored, queried, edited, and visualized.

### MongoChemServer: server side platform

The server code was developed using the Python 3 language as a Girder plugin. The Girder framework is an open source project, and released under the Apache 2.0 license. It has three main components:

- Data organization and dissemination
- User management and authentication
- Authorization management

It is developed as an extensible data management platform, and it reuses a number of open source projects including CherryPy—“a pythonic, object-oriented web framework”. The code in the mongochemserver repository [30] extends the functionality provided in a plugin that is loaded by the Girder process when it starts up. The plugin adds RESTful API, reuses core functionality and core plugins for more generic features such as authentication, Gravatars, file upload/download, access control, etc.

The platform provides integration with MongoDB, using that to store user credentials, access permissions, metadata, and other elements exposed via its plugin system. Among the most useful abstractions provided in the context of this project are the authentication, access permission, and file storage systems. Almost all of these concepts must be exposed on both the server and in the web client code to be used effectively.

The existing OAuth2 plugin was used, and coupled with Google’s OAuth2 implementation to offer single-sign on. This can be replaced with other authentication schemes, or augmented with multiple options. For simplicity this was the only option offered in the prototype described, coupled with the use of encrypted SSL connections to provide secure authenticated access. This choice enabled the deployment of a demonstration to multiple locations, but was not always the most appropriate and will be augmented in future development to include integration with site-wide systems where appropriate.

**Fig. 2:**
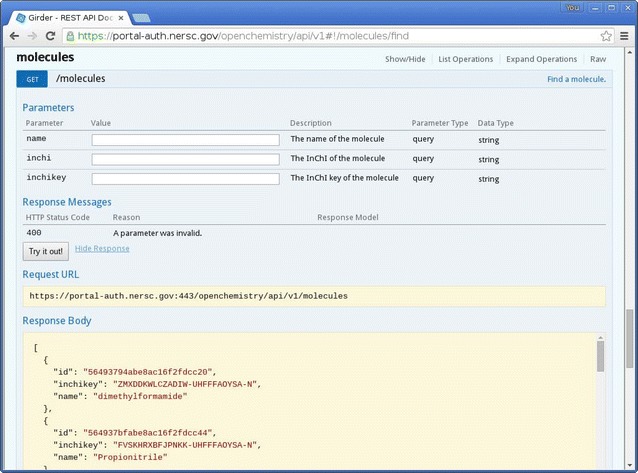
Swagger documentation for RESTful API. An example of Swagger being used to test some of the RESTful API in the ‘molecules’ resource

The access permissions can be applied at several levels in the code. A RESTful API must be exposed as a resource which resolves various paths, which refer to namespaces within the API prefix and are documented using a system called “Swagger”. This enables developers to document API as it is written, provides an HTML5 web client that exposes this documentation, and offers the ability to test API live on the web, shown in Fig. 2. The API exposed uses decorators to express whether a given piece of API is public, or can only be accessed by authenticated users. API that requires authentication can apply further restrictions based on user privileges, and provide filtered results containing only data the authenticated user has the access privileges for.

File upload/download sounds quite simple on the surface, but it involves a number of distinct components in order to scale and integrate well in different environments. The project uses asset stores to abstract the storage backend, and the backend can then be mapped to file systems, S3 storage (as provided by Amazon EC2), and others. Large files must also be uploaded/downloaded in “chunks”, something offered as part of the standard file API and exposed in the client application. File system storage proved sufficient for the work described, but future deployments would benefit from using large file stores, with extension to archive servers at supercomputing centers.

The web APIs were extended with three main endpoints for the chemical data server. The “molecules” prefix provides functions to interact with molecular data, and is linked to from the other objects created. A molecular graph is unique, and other objects such as calculations will refer to a molecule. The simplest way to use the molecules endpoint is to use a GET query on the name, InChI, or InChI key of a molecule to see if it exists. If the search results in a math then a JSON array will be returned with objects containing the fields id, inchikey, and name that can be used to retrieve each molecule. If no match then an empty JSON array will be returned.

Retrieving molecule records can be achieved using the molecules/id endpoint, using the URL “/api/v1/molecules/564a2fdd5573c07ff61ce3db/xyz” would retrieve the molecule with the ID of “564a2fdd5573c07ff61ce3db” in the XYZ format. Changing that to “/api/v1/molecules/564a2fdd5573c07ff61ce3db/cml” would retrieve the same molecule in the CML format, and ending it with ’cjson’ would return the molecule in the Chemical JSON format described later. There is a similar endpoint to query the database using InChI keys. A “/molecules/conversions/{output_format}” endpoint provides file format conversion services.

A “/molecules/search” endpoint offers a simple query language that was exposed in the web interface, where it is possible to search on molecular mass with numeric comparisons, logical AND or OR queries together. It contained a number of string based values that could be searched on, such as InChI, InChI key, name, chemical formula, as well as numeric fields such as mass, atom count, and heavy atom count. This was implemented in a Python file, and exposed in the endpoint, with inline documentation available by clicking on the search icon.

The other major endpoint developed was the “calculations” prefix, this provides access to quantum chemical calculations. They must have a parent molecule, and are primarily accessed using the GET method, querying on the “moleculeId”. If there are calculations present for a given moleculeId, a JSON array will be returned with objects containing the “_id” (the identifier for the calculation), with various properties such as the name of the code performing the calculation, the theory, calculation types contained, and a file identifier that refers to the original output used to build the record. The calculation identifier can then be used to retrieve other elements of the calculation, such as the atomic coordinates as a JSON file, a cube for a given molecular orbital, vibrational modes, etc.

All of these endpoints, along with the more generic ones usch as file, folder, group, item, user, etc can be viewed using the built in Swagger. One of the important results is not the features described, but the simple mapping of Python code to endpoints, with inline documentation, that can be rapidly extended and deployed. Python offers an excellent glue, providing easy access to native Python code, C/C++ wrapped libraries, and even other web services.

### MongoChemClient: rich HTML5 web client

The Girder project has its own web interface, but this was not used—a custom user interface was developed in the mongochemclient repository [31]. The interface developed in this repository is a modern HTML5 web interface. This means that all HTML5 assets can be served as static files, and the page is built up dynamically on the client-side using the web API to authenticate (if necessary), retrieve data, upload new date, and visualize data. A number of technologies and projects were leveraged in order to create a compelling, modern interface in a relatively short space of time.

**Fig. 3:**
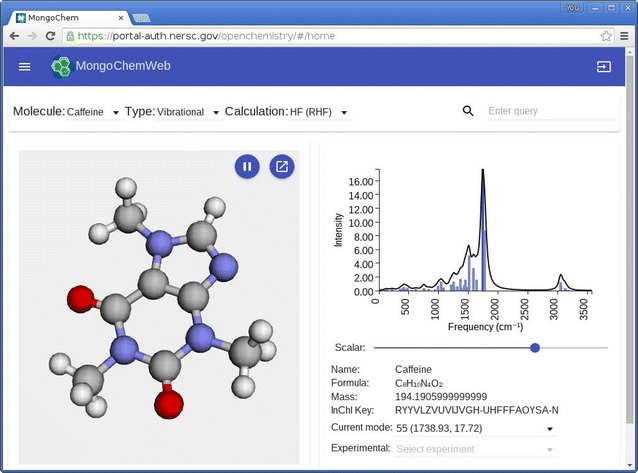
HTML5 web client with molecular structure and vibrational modes. The web client displaying a molecular structure in 3DMol.js, and selection of vibrational modes in a D3 chart

The main framework used to manage interaction, react to events, and coordinate the single dynamic page approach was AngularJS. This framework is divided into a number of modules that provide various services/extensions, such as easy access to APIs, animations, routing (where the URL is updated to reflect the current ‘location’ (state) despite being in a single-page web application), and overall look and feel (Materials design in this instance). AngularJS was chosen for the rich feature set, maturity, and encapsulation of components along with its powerful web page layout framework. An example of the single page application in action is shown in Fig. 3, where a molecular structure can be seen beside a plot of vibrational modes that can be animated in the 3DMol.js based geometry viewer.

The static HTML5 content that serves as the frontend, and the dynamic programming interfaces offering access to the data must be presented to the web client. This is where the NGINX web server [32] came in, offering an SSL-enabled endpoint for encryption, serving the static content in the web root, and proxying requests to the /api/v1 prefix to the Python-based backend. The Python-based backend also had access to the MongoDB server where all metadata, access controls, and links to files were stored. There is also an asset store, where a simple on-disk asset store was used.

The software infrastructure involves a number of build/deployment systems, and a deployment repository [33] was necessary to coordinate the task of deploying everything to the right location, with compatible software versions, and ensuring services are brought up/down in the correct order. The project employed an industry standard open source tool called Ansible [34] to document how the service was deployed on Amazon’s EC2 infrastructure, and this approach can be adopted to other environments. Ansible automates the process of logging into specified web hosts, setting up users, installing packages, and placing everything in the correct place before starting services such as the database and web services.

### JSON data formats

Over the years, various data formats have been developed for computational chemistry to enable, among other things, information exchange and visualization. More recent work has focused on the development of ways to express results (log files) from computational chemistry in data formats that are suitable for knowledge discovery on the semantic web. One example is the XML based Chemical Markup Language (CML) [35].

Here, we chose to build a data format using JSON because it is less cumbersome than XML, with simpler parsers, and is a native format of the web. It is based on many of the concepts developed in the CML format as part of several ongoing collaborations under the broad umbrella of the Blue Obelisk [36, 37]. Two JSON formats were developed in parallel, one focused on providing information essential for visualization platforms, and one with the goal to provide semantically enriched information from computational chemistry codes as an alternative to plain text log files. Comparison of the two JSON formats provides insight into the disparate needs of the use cases, and can serve as a guide for the development of a community wide JSON data format.

The Chemical JSON format within the Avogadro 2 project has been in development for a number of years, with the focus on providing a simple means of data storage and exchange of chemical data for visualization. The JSON format was developed based upon the requirements of storing data in BSON using MongoDB, communicating chemical data over JSON-RPC 2.0 between desktop applications, and later using web services. It was also motivated by the need for a format to support an application being developed to edit and communicate chemical structures. The format was tested with small molecules through to molecules with millions of atoms using a philosophy of being machine readable, efficient, and avoiding repetition. It was written to represent a simple mapping of the in memory data structures.

This JSON format exposed the internal data model used in Avogadro 2, and was used as the transport layer to the 3DMol.js client-side rendering. It was extended to expose vibrational modes, and a primitive container for volumetric data already present in the 3DMol.js project. The current state of the Chemical JSON format is documented in a Github repository [38], and will continue to evolve as it is extended to serve more use cases.

In parallel, the ChemLog JSON format was developed with the goal of encapsulating the data from computational chemistry software, such as the open source NWChem, with semantic annotation. The goals of the two formats were somewhat orthogonal. Chemical JSON expresses the properties of a single molecule in machine readable arrays that contain the essential data that might be visualized/displayed. In contrast, the ChemLog JSON format is focused on storing all the essential input and output data with semantic annotation from the computational chemistry software simulation (here for NWChem) for the (possibly multiple) simulation(s). This is somewhat akin to the typical molecular file formats (XYZ, CML, SDF, etc) versus a more structured quantum mechanical log file—a snapshot of definite structure versus data discovery and recording.

This new format focuses on satisfying the need to replace a log file with structured output, offering output of multiple calculations as a computational chemistry code like NWChem is executed. The JSON file generated by the NWChem application builds upon previous work done integrating CML into NWChem [39] (an XML-based format), with some related efforts extending CML for the semantic web using an intermediary format called CSX [40]. It is designed around the notion of using objects as the primary representation. As a starting point the CML naming and conventions were adopted and extended with new concepts. The format was developed and designed to be portable to multiple computational chemistry codes but in this work firmly targeted the NWChem code.

The relevant section representing the geometry/structure of JSON files generated in the two formats, in this case for a water molecule, are compared to clearly show the difference in design philosophies, i.e. arrays vs objects, driven by the use case, i.e. visualization vs semantically enriched semantic. The listing below shows a molecule block within the Chemical JSON format:

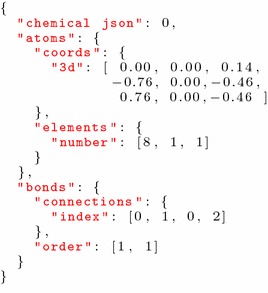



The format focuses on using arrays to store information about atoms, bonds, etc—such as the atomic number, bond order, etc. The 3D coordinates are in an array, and each 3D position is offset by 3N where N is the index of the atom. This offers compact, simple representations with efficient storage of atomic data. It does not provide a layout that is focused on human readability, and it is not intended to be used as a directly editable set of objects in Python/JavaScript without some code in front of it to aid in manipulation/keeping the representation consistent. This representation is very easy to use in web platforms where asynchronous network calls can bring in progressively more data about the molecule, starting with just atoms, then bonds, then molecular orbital cubes, vibrations, etc.

The listing below shows the same molecule block within the ChemLog JSON format:

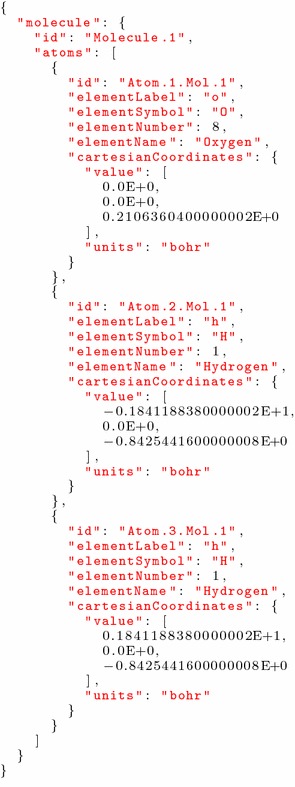



In contrast to the Chemical JSON listing, all information and properties of each atom in ChemLog JSON is collected in a single object. A key aspect of this format is the explicit definition of units, a key aspect brought in for the CML specification. It is relatively simple to map between the two formats, and this is now possible in the Avogadro 2 libraries which feature readers for both formats.

In developing the ChemLog JSON format, some additional referencing features were introduced that are not natural to JSON. The listing below shows an excerpt of a JSON data file containing the calculation setup and some of the properties that can be calculated with quantum chemistry software:

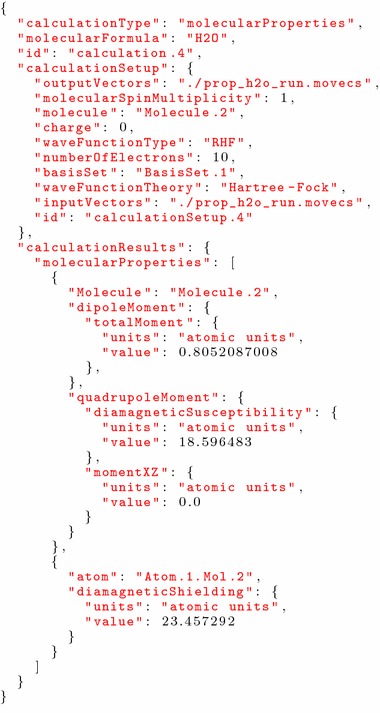



Input structures in computational chemistry codes are set up to allow a sequence of calculations to be executed in one single run. To store this sequence, the ChemLog JSON format has adopted the CML approach of storing each calculation in a “calculations” array. The listing above shows an excerpt of the fourth calculation in this series. Often, data from previous calculation steps in the sequence is reused in the quantum chemistry code. To avoid duplicating information, “id” tags were used to provide a pointer to the first mention of the JSON object. An example of this in the listing above are the key “molecule” to “Molecule.2”, which is defined as the id-tag in the molecule block in the previous listing. Another example is the “basisSet” keyword in the “calculationSetup” block. Even the “calculationSetup” could be the same for multiple calculations, and could be pointed to in this fashion. The same approach is used to identify the atom within the molecule to which the calculated atomic properties belong. Here the “atom” keyword points to atom “Atom.1.Mol.2” in the earlier ChemLog JSON listing for the molecular geometry. Essentially, this referencing approach mimics the ability to link different sets of data. The referencing feature is missing in the current JSON specification but native to XML. The JSON pointer has been proposed in RFC 6091 [41] and a draft RFC is being developed for the JSON reference [42]. While JSON reference would provide the necessary flexibility, either could readily be adopted in the format in the near future.

Examples of complete ChemLog JSON files generated by the NWChem quantum chemistry code can be found on the Github repository [29].

The JSON file can be submitted to the web platform developed, and relevant metadata will be extracted. The format is capable of encapsulating jobs that have multiple steps, and it has been demonstrated with vibrational and electronic structure data. The format breaks most elements of the output into distinct objects, and it has been designed to be more semantically expressive than the Chemical JSON format developed as part of the Avogadro 2 project. Future work will draw upon new developments in JSON-LD [40, 43] that aims to bring linked data and meaning to JSON using the same approaches used in semantic data structures.

The two formats were used as part of this project, with several extensions made to the Chemical JSON format in order to support data needed by the project. They are both supported by the Avogadro 2 libraries, and exposed in the Python-wrapped API used on the server side. The Chemical JSON format is designed in a pragmatic fashion, making extensive use of arrays, along with key-value pairs for simple properties. The format closely matches in-memory structures, it is optimized for storage in BSON (the binary form of JSON used internally by MongoDB), is easy to visualize and store as a single document in MongoDB. The new ChemLog JSON format developed tends to represent everything as an object, can store multiple configurations/states of a molecule, and uses links to reuse objects that have not changed. This makes it more complex to parse, but more expressive, and it more closely reflects what is currently stored in log files.

As stated before, extensions were needed in the internal models used in the Avogadro 2 projects, the 3Dmol.js project, and in the model employed in the web framework. Ultimately, any given visualization can only show a single state, and the web and desktop frameworks both generally assume a file only has a single state with minimal links back to the input file. This concept will need to be extended in order to visualize and analyze data from more complex multi-step files, and some of this development was started in the work described.

## Discussion

Research in chemistry is becoming more data intensive, and as a result it is vital that we act to create platforms that can be easily deployed, used, and shared with a focus on data. This requires standard formats that support a multitude of facets of chemical data that leverage industry standard technologies such as XML, JSON, and the semantic web.

As we move closer to a landscape where the web is an essential part of any workflow we must embrace web-native formats, generally using JSON and similar containers, to enable the free exchange, analysis and visualization of data. This can be further augmented through the use of JSON-LD, and is something the authors would like to do in order to offer semantic meaning and enable the simple transformation of JSON-LD documents to triples.

The use of triples open up the semantic, linked web that is becoming increasingly important as this widens access to the data. As we move forward, and data-centric computing proliferates embracing the standards of the semantic web will make it easier to find and use the data generated, and it will also make the meaning of specific keys clear to machines, i.e. the definition of a 3D coordinate, and the units it is expressed in, of the “energy” of a quantum calculation—what quantity is being expressed, on what molecule, and in what units. This requires normalized data, but goes well beyond it to express data less ambiguously and more formally.

Once these components are available the open source code referenced here, and available as a demonstration capability, show what can be built around it. The RESTful APIs, databases, and HTML5 frontends can be readily coupled with command line and powerful desktop applications to offer a compelling ecosystem of data-centric applications that serve the chemical research enterprise.

The definition of suitable schema, codification of that into JSON-LD, and community agreement still represent significant barriers to wider adoption. The development of permissively licensed open source code, which can be readily inspected, reused, and modified is one approach to improving the situation. Coupled with the use of community platforms such as GitHub for collaborative development, and workshops gathering interested experts, these approaches can be iterated upon and standardized.

## Conclusions

A “full-stack” open source web application was developed, making heavy use of Python and open source community tools on the backend, and HTML5/JavaScript on the frontend. Years of experience in using, converting, and developing data formats was drawn upon to create some new formats for the output of structured data from the NWChem code, ingestion into databases, and the subsequent visualization/analysis of data. This was demonstrated in a new web client, and added to the Avogadro 2 desktop application. The primary data types were 3D chemical structure, electronic structure, and vibrational modes on the web and desktop.

If the field of chemical sciences is to progress towards knowledge discovery on the semantic web, it is important to move away from disparate formats and basic data log files. The ingestion and normalization of data still represents a serious challenge to reaping the benefits powerful data-centric platforms promise. The Avogadro project provides translation to and from standard chemical data formats, these can be read by Open Babel and translated to all supported formats. The authors were made aware of a new effort by the European Materials Modeling Council (EMMC) on enhancing interoperability in materials modeling codes. As this paper was written, the authors and researchers at the Molecular Sciences Software Institute (MolSSI) started a working group committed to the development of a unified community (web, visualization, workflow, computational chemistry codes, and others) supported JSON data format.

## Abbreviations

3D: three dimensional
API: application programming interface
CSS: cascading style sheets
CML: Chemical Markup Language
HTML: Hypertext Markup Language
HPC: high-performance computing
JSON: JavaScript object notation
LBNL: Lawrence Berkeley National Laboratory
REST: representational state transfer
SSL: Secure sockets layer
XML: Extensible Markup Language
